# Clausenidin induces caspase-dependent apoptosis in colon cancer

**DOI:** 10.1186/s12906-016-1247-1

**Published:** 2016-07-29

**Authors:** Peter M. Waziri, Rasedee Abdullah, Swee Keong Yeap, Abdul Rahman Omar, Nur Kartinee Kassim, Ibrahim Malami, Chee Wun How, Imaobong Christopher Etti, Mary Ladidi Abu

**Affiliations:** 1 MAKNA Cancer Research Laboratory, Institute of Bioscience, University Putra Malaysia, Serdang, Selangor, Malaysia; 2 Department of Biochemistry, Kaduna State University, Main Campus, PMB 2336 Kaduna, Nigeria; 3 Department of Veterinary Pathology and Microbiology, Faculty of Veterinary Medicine, University Putra Malaysia, Serdang, Selangor, Malaysia; 4 Laboratory of Vaccine and Therapeutics, Institute of Bioscience, University Putra Malaysia, Serdang, Selangor, Malaysia; 5 Department of Chemistry, Faculty of Science, University Putra Malaysia, Serdang, Selangor, Malaysia; 6 Enzyme Technology Research Laboratory, Institute of Bioscience, University Putra Malaysia, Serdang, Selangor, Malaysia

**Keywords:** Clausenidin, Apoptosis, Colon cancer, MMP, Caspase 9, bcl 2, bax

## Abstract

**Background:**

*Clausena excavata* Burm.*f*. is a shrub traditionally used to treat cancer patients in Asia. The main bioactive chemical components of the plant are alkaloids and coumarins. In this study, we isolated clausenidin from the roots of *C. excavata* to determine its apoptotic effect on the colon cancer (HT-29) cell line.

**Method:**

We examined the effect of clausenidin on cell viability, ROS generation, DNA fragmentation, mitochondrial membrane potential in HT-29 cells. Ultrastructural analysis was conducted for morphological evidence of apoptosis in the treated HT-29 cells. In addition, we also evaluated the effect of clausenidin treatment on the expression of caspase 3 and 9 genes and proteins in HT-29 cells.

**Result:**

Clausenidin induced a G0/G1 cell cycle arrest in HT-29 cells with significant (*p* < 0.05) dose-dependent increase in apoptotic cell population. The DNA fragmentation assay also showed apoptotic features in the clausenidin-treated HT-29 cells. Clausenidin treatment had caused significant (*p* < 0.05) increases in the expression of caspase 9 protein and gene in HT-29 cells and mitochondrial ROS and mitochondrial membrane depolarization. The results suggest the involvement of the mitochondria in the caspase-dependent apoptosis in clausenidin-treated colon cancer cells.

**Conclusion:**

Clausenidin induces a caspase-dependent apoptosis in colon cancers through the stimulation of the mitochondria. The study demonstrates the potential of clausenidin for use in the treatment of colon cancers.

## Background

Colon cancer is the second leading cause of cancer deaths worldwide accounting for over 1.4 million new cases each year [1, 2]. Colon cancers are rising at an alarming rate in countries where very few treatment options are available. It is projected that from 2015 to 2030 the incidence of the disease in developing countries will increase by as much as 60 % [3] and will present great challenges to the management of the cancer. It has been shown that there are close associations between molecular signaling, energy generation, and proliferation of tumors [4, 5]. Since these molecular mechanisms can promote tumor cell proliferation, they are usually the targets of cancer chemotherapies [6].

Cancer chemotherapeutic agents are screened and selected based on their potentials to induce apoptosis [7]. Apoptosis is an integral cell death program characterized by DNA fragmentation as well as loss of mitochondrial membrane integrity [8]. This mode of cell death is driven by caspases and proceeds via the extrinsic or intrinsic pathways. The intrinsic pathway of apoptosis is tightly coordinated by the mitochondria via the activation of caspase 9 [9, 10]. Because of the integral role it plays, caspase 9 is often referred to as gatekeeper of the mitochondrial pathway [11]. Upon activation, caspase 9 activates caspase 3, whose action leads to the execution of apoptosis. Apoptotic strategies to destroy tumor cells may involve direct stimulation of pro-apoptotic molecules, modulation of anti-apoptotic proteins or induction of tumor suppressor function [12]. The functions of caspase 9 gene and promoter help to drive apoptosis to ensure that abnormal and senescent cells are removed from the body [13]. A comparison between the normal and tumor cells of colorectal cancer patients had shown decreased expression of caspase 9 in tumors [14]. In situations where tumors arise due to altered gene expression, treatments can be targeted towards improving the expression of the gene because the activation or inhibition of certain genes could stimulate apoptosis and alter the kinetics of tumor growth [15].

**Fig. 1:**
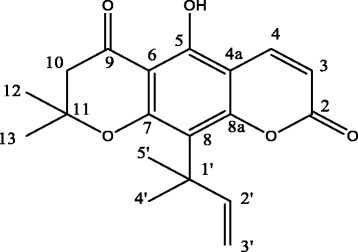
Structure of Clausenidin

Clausenidin (Fig. 1) is a natural pyranocoumarin from *Clausenidin excavata* Burm*. f.*, a wild shrub of the *Rutaceae* family predominantly used in Asian folk medicine [16, 17]. The plant has been used locally to treat cold, dermatopathy, snake bite, malaria, HIV and abdominal pains [18, 19]. In Thailand, it has been reported that *Clausena excavata* is traditionally used in the treatment of cancers [17]. In a recent study, Su et al. [20] isolated four pyranocoumarins from *C. excavata* and screened their cytotoxic potentials in cancer cells. The study revealed that the pyranocoumarins are good modulators of tumor cell death. In another study dentatin isolated from *C. excavata* was shown to increase the expression of caspase 9 in MCF-7 cells [21]. In our laboratory we observed that the expression of caspase 9 increases in clausenidin-treated hepG2 cells (Unpublished report). However, the underlying mechanisms by which clausenidin induces apoptosis has not been fully resolved. The current study describes for the first time, some molecular mechanisms involved in clausenidin-induced cell death in a colon cancer cell line. The study also provides insights on caspase-dependent apoptosis triggered by clausenidin in colon cancers.

## Methods

### Extraction and isolation of compound

Fresh roots of *Clausena excavata* Burm.*f*. were collected from the Agricultural Park, Institute of Bioscience, University Putra Malaysia. The identification of the plant material was done by Dr Shamsul Khamis of the Institute and a voucher specimen was deposited in the herbarium (2991/16). The roots (2 kg) were air-dried for 2 weeks, ground to fine powder and used for the extraction process. The extraction of clausenidin was performed by soaking the fine powder in 1 L chloroform for 3 days followed by filtration through 0.45 μm filter paper (Sigma Aldrich, US) to collect the extract. The extract was subjected to glass column chromatography through silica gel, as described by Arbab et al. [21], to obtain pure clausenidin crystals (524 mg). The clausenidin crystals were subjected to mass spectrometry (Shidmadzu GCMS-QP5050A) while the melting point was determined using a Barnstead melting point apparatus [16, 18].

### Cell viability assay

Human colon cancer (HT-29) cells were obtained from American Type Culture Collection (ATCC, Va, USA) and doxorubicin from EMD Millipore, US. The cells were maintained in DMEM medium supplemented with 10 % Fetal Bovine Serum (FBS). About 5000 cells were seeded into each well of a 96 well plate and incubated for 24 h. Treatment was done with increasing concentrations of clausenidin and doxorubicin (positive control). Negative control cells were treated with 0.1 % (v/v) DMSO. After 72 h, MTT (5 mg/mL) was added for the viability assay as described previously by Syam [22]. Results obtained were expressed as percentage cytotoxicity after 72 h exposure to test agents.

### Morphological assessment of apoptotic cells by acridine orange (AO) and propidium iodide (PI) double staining

Clausenidin induced cell death in HT-29 cells was monitored using acridine orange (AO) and propidium iodide (PI) double-staining according to standard procedures. Cells were seeded in a 6 well plate (10^5^cells/well) and incubated overnight. The cells were treated for 24 h with increasing concentrations of clausenidin after which the cells were harvested and washed with PBS. The cells were centrifuged at 1000 g for 5 min and the supernatant was discarded. The washing procedure was repeated twice to remove traces of media from the cells. Ten microliters (10 μl) of fluorescent dyes containing AO (10 mg/mL and PI (10 mg/mL) were added into the cellular pellet at equal volumes. Freshly stained cell suspension was dropped on a glass slide and covered with a cover slip. Slides were observed under fluorescent microscope within 30 min before the fluorescence fades.

### Transmission electron microscopy

To prepare the cells for transmission electron microscopy (TEM), HT-29 cells were seeded at a density of 10^6^cells/T-25 ml flask and incubated overnight. The cells were then treated with the IC_50_ of clausenidin (13.8 μg/mL) in a time dependent manner while the negative control cells were treated with 0.1 % (v/v) DMSO. After treatment, cells were harvested and washed with PBS before successive fixing with 4 % glutaraldehyde for 24 h and 1 % osmium tetraoxide at 4 °C for 2 h. After each fixing, washing was done three times with 0.1 M sodium cacodylate buffer. Dehydration of the cells was carried out with increasing concentrations of acetone (30 – 99.9 %). Further processing of cut sections was done using the method described by Tan et al. [23]. The sections were stained with uranyl acetate and viewed under the Hitachi H-7100 electron microscope.

### ROS assay

The ROS assay was performed to measure the intracellular ROS production from the mitochondria of clausenidin-treated HT-29 cells. Briefly, cells were seeded at a density of 2 × 10^5^ cells/well in a 6 well plate and incubated overnight. The cells were then treated with clausenidin (13.8 μg/mL) at increasing time period and harvested for the ROS assay, which was performed using the Total ROS assay kit (ebioscience Inc, Affymetrix) according to manufacturer’s protocol. The assay results were analyzed on a flow cytometer (BD FACS, Calibur).

### DNA fragmentation analysis

The HT-29 cells were seeded at a density of 10^6^cells/ml in culture flask and incubated overnight. The cells were then treated with clausenidin (13.8 μg/mL) for 12 h and 24 h respectively after which the cells were harvested and DNA was extracted using the suicide-track™ DNA Ladder Isolation kit (Calbiochem, USA) according to the manufacturer’s protocol. The principle involves detection of the cytoplasmic histone-associated DNA fragments (mononucleosome and oligo- nucleosomes) formed during apoptosis. After extraction, the concentration and purity of the DNA was confirmed on a nanodrop spectrophotometer. The extracted DNA samples were run on a 1.5 % agarose gel in Tris–acetic acid–EDTA buffer and gel image was captured on GelDoc (Biorad, USA). HL-60 cells induced to undergo apoptosis with Actinomycin D, supplied with the assay kit was used as positive control.

### Cell cycle analysis by flow cytometry

The HT29 cells were seeded at a concentration of 10^6^cells/T-25 ml flask in RPMI media and incubated overnight. The cells were then treated with clausenidin (13.8 μg/mL) at an increasing time period while the negative control cells were treated with 0.1 % (v/v) DMSO. After treatment, cells were harvested and washed with PBS. The cell cycle assay was performed using BD cell cycle reagent (CycleTest™ Plus DNA reagent kit, Becton Dickinson, Belgium) according to manufacturer’s protocol and the result was analyzed on a flow cytometer (BD FACS, Calibur) using the BD cell quest pro software.

### Annexin V assay

HT-29 cells were seeded at a concentration of 2 × 10^5^cells/T-25 flask in RPMI media and incubated overnight. The cells were then treated with increasing concentrations of clausenidin while the negative control was treated with 0.1 % (v/v) DMSO. Cells were harvested after treatment and washed with PBS. Annexin V assay was then carried out using FITC annexin V assay kit (BD Pharmingen, USA) following the manufacturer’s protocol and the result was analyzed on a flow cytometer.

### Mitochondrial membrane potential (MMP) assay

Mitochondrial membrane potential assay was performed to determine the polarization/depolarization of the mitochondrial membrane using the JC-1 dye. HT29 cells were seeded at a density of 5 × 10^5^cells/well in a 6 well plate, incubated overnight and then treated with increasing concentrations of clausenidin for a period of 24 h. The negative control cells were treated with 0.1 % (v/v) DMSO. The assay was performed using the BD™ Mitoscreen kit (BDbiosciences, US) according to manufacturer’s instruction. The results were analyzed on a flow cytometer (BD FACS, Calibur) using the BD cell quest pro software.

### Caspases 3 and 9 assays

Caspases 3 and 9 activity were determined using the colorimetric method (Genescript Colorimetric Assay kit, USA). Cells were initially seeded in a 6 well plate at a density of 10^6^ cells/well overnight. The cells were treated for 24 h with increasing concentrations of clausenidin and then cleaved Caspases 3 and 9 assays were performed respectively according manufacturer’s protocol. Negative control cells were treated with 0.1 % (v/v) DMSO. After the completion of reaction, the plates were read on microplate reader at 405 nm.

### RNA isolation

RNA extraction was basically done to study the expression of the genes and get possible insights into the mechanism of apoptosis induced by clausenidin. After treatment of cells with clausenidin and 0.1 % DMSO (for negative control) in a 6 well plate (10^6^cells/well), the cells were harvested with trypsin and washed with PBS. The RNA was extracted using the Total RNA extraction kit (GF-1 TRE kit, Vivantis technologies) according to the manufacturer’s protocol. The extracted RNA was quantified using a nanodrop spectrophotometer at 260 nm.

### RT-qPCR

The reverse transcriptase quantitative PCR (RT-qPCR) was carried out according to the GenomeLab GeXP Kit (Beckman Coulter, USA) protocol, in an XP Thermal Cycler (Bioer Technology, Germany). PCR products were finally analyzed on the GeXP genetic analysis system and the results normalized on express Profiler software based on the manufacturer’s instructions. The primers for the genes of interest and housekeeping gene (Table 1) were designed on NCBI website and purchased from Biosune (Shanghai, China), while the internal control (Kanr) was supplied by Beckman Coulter (USA).

**Table 1:** Gene name and sequences of primers used in the multiplex panel

Gene Name	Forward sequence	Reverse sequence
Bax	AGGTGACACTATAGAATAGCAAACTGGTGCTCAA	GTACGACTCACTATAGGGAAACCACCCTGGTCTTG
Bcl-2	AGGTGACACTATAGAATACTGTGGATGACTGAGTACCT	GTACGACTCACTATAGGGATCAGAGACAGCCAGGAG
Apaf-1	AGGTGACACTATAGAATACATACTCTTTCACCAGATCA	GTACGACTCACTATAGGGAACAAGTTCTGTTTTTGCTTT
Cyt c	AGGTGACACTATAGAATAGAGCGAGTTTGGTTGC	GTACGACTCACTATAGGGAAAATCTTCTTGCCTTTCTC
Caspase 3	AGGTGACACTATAGAATATGTAGAAGAGTTTCGTGAGT	GTACGACTCACTATAGGGAGAGTTTTCAGTGTTCTCCAT
Caspase 9	AGGTGACACTATAGAATAGCTGGTGGAAGAGCTG	GTACGACTCACTATAGGGACTCTAAGCAGGAGATGAACA
β-actin	AGGTGACACTATAGAATAGATCATTGCTCCTCCTGAGC	GTACGACTCACTATAGGGAAAAGCCATGCCAATCTCATC

The β-actin gene was used for normalization. Reverse transcription (RT) and PCR were done according to manufacturer’s instructions; RT reaction was at 480 °C for 1 min; 370 °C for 5 min; 420 °C for 60 min; 950 °C for 5 min; then held at 40 °C, while PCR was as follows: initial denaturation at 950 °C for 10 min, followed by two-step cycles of 940 °C for 30 s and 550 °C for 30 s, ending in a single extension cycle of 680 °C for 1 min. Bax: Bcl-2-associated X protein; Bcl-2: B cell lymphoma 2; Apaf-1: Apoptotic protease activating factor 1; Cyt c: cytochrome complex; Caspase 3: Cysteine aspartic acid protease 3; Caspase 9: Cysteine aspartic acid protease 9

### Statistical analysis

Data were presented as mean ± standard deviation. One way Analysis of Variance (ANOVA) on SPSS 22 software (SPSS Inc, Chicago IL, USA) was used to assess the level of significance between means at 95 % confidence interval (*p* < 0.05).

## Results

### Cell viability and cytotoxicity assay

**Fig. 2:**
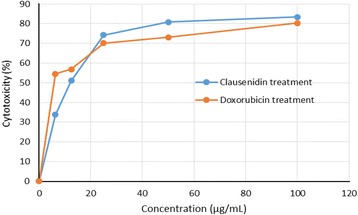
Cytotoxicity of HT-29 cells treated with clausenidin and doxorubicin. The IC_50_ of clausenidin and doxorubicin is 13.80 ± 2.89 and 6.20 ± 0.14 μg/mL, respectively

The cell viability assay was performed to evaluate the percentage (%) cytotoxicity and IC_50_ of pure clausenidin in HT-29 cells. Clausenidin induced cytotoxic effects in HT-29 cells in a dose dependent manner (Fig. 2). The IC_50_ of clausenidin obtained after treating the cells for 72 h is 13.8 ± 2.89 μg/mL while that of doxorubicin is 6.2 ± 0.14 μg/mL.

### Fluorescent microscopy

**Fig. 3:**
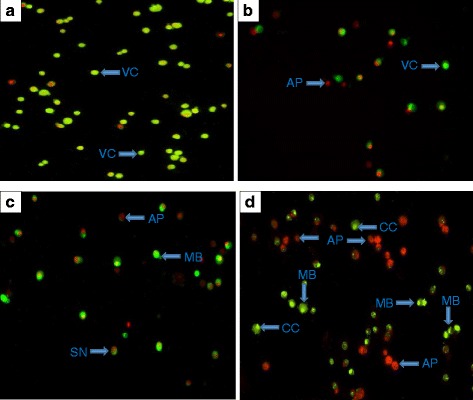
Acridine orange and propidium iodide-stained clausenidin-treated HT-29 cells. **b**, **c**, **d** are treatments with 5, 15, and 30 μg/mL clausenidin, respectively and (**a**) is the untreated control. VC - Viable cells, AP - apoptosis, MB - membrane blebbing, SN - necrosis. Analyses in triplicates. *Significant difference between means at *p* < 0.05

The fluorescent microscopy was performed after staining the clausenidin-treated cells with equal proportions of acridine orange and propidium iodide dyes. The fluorescent micrograph reveals the apoptosis inducing effects of clausenidin in HT-29 cells (Fig. 3). Some of the morphological aberrations observed includes membrane blebbing and chromatin condensation which are early signs of apoptosis (Figs. 3b - d). In addition some cells were observed to be undergoing secondary necrosis.

### Transmission electron microscopy

**Fig. 4:**
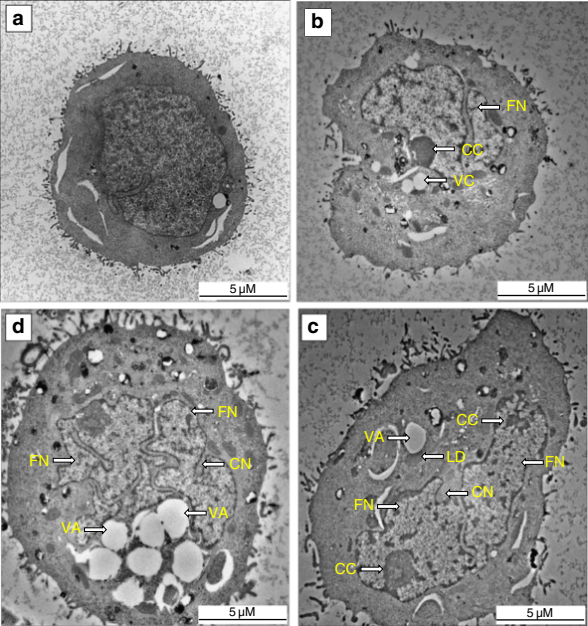
Ultrastructural assessment of clausenidin-treated HT-29cells. **b**, **c**, **d** are treatments (13.8 μg/mL) for 24 h, 48 h, and 72 h respectively and (**a**) is the untreated control. VA - vacuole; LD - lipid droplet; FN - fragmented nucleus; CC - chromatin condensation; CN - convolution of nuclear outline. Analyses in triplicates

To further confirm the occurrence of apoptosis, the ultrastructural micrograph of clausenidin treated HT-29 cells were monitored in a time dependent manner and it revealed the presence of nuclei shrinkage and fragmentation, chromatin condensation, lipid droplets and convolution of nuclei outline which are hallmarks of apoptosis (Fig. 4). An increase in vacuolation and loss of microvilli was observed as the treatment time increased.

### ROS production assay

**Fig. 5:**
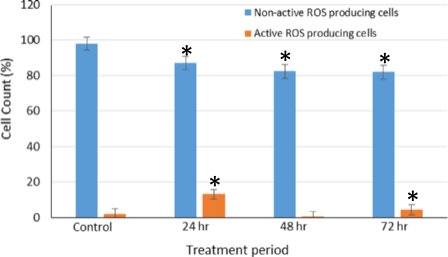
Reactive oxygen species production by clausenidin-treated HT-29 cells. Analyses in triplicates. *Significant difference between means at p < 0.05

The production of ROS in HT-29 cells was measured using flow cytometry in the FITC channel. The assay method employed identified only cells that are actively producing the intracellular ROS. The result in Fig. 5 shows that the percentage (%) of HT-29 cells producing ROS increased significantly (*p* < 0.05) after treatment with clausenidin at 24 h compared to the untreated cells. However, a decline was observed at 48 and 72 h compared to that of 24 h because the active ROS producing cells were dying due to the oxidative stress (Fig. 5c and d). Conversely, the percentage of the non-active ROS producing cells in the treatments decreased significantly (*p* < 0.05) as the treatment time progressed. This is due to stimulating effect of clausenidin which caused the more cells to begin the production of ROS.

### DNA fragmentation analysis

**Fig. 6:**
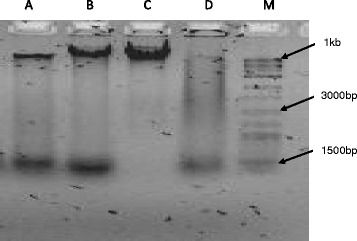
DNA fragmentation in clausenidin-treated HT-29 cells. A and B are treatments (13.8 μg/mL) for 12 h and 24 h respectively, D is positive control supplied with the kit while C is the untreated control. M is the molecular marker. Analysis in triplicates

The HT-29 cells were treated with clausenidin (13.8 μg/mL) at 12 h and 24 h which led to the generation of DNA fragments shown in the gel image (Fig. 6). The untreated cells (negative control) had their genomic DNA intact with no fragments generated (lane C) unlike the treatments (lanes A & B) and positive control (lane D). Clausenidin induced an internucleosomal cleavage of DNA in HT-29 cells leading to the generation of fragments which is a basic feature of apoptosis [24, 25].

### Cell cycle analysis

**Fig. 7:**
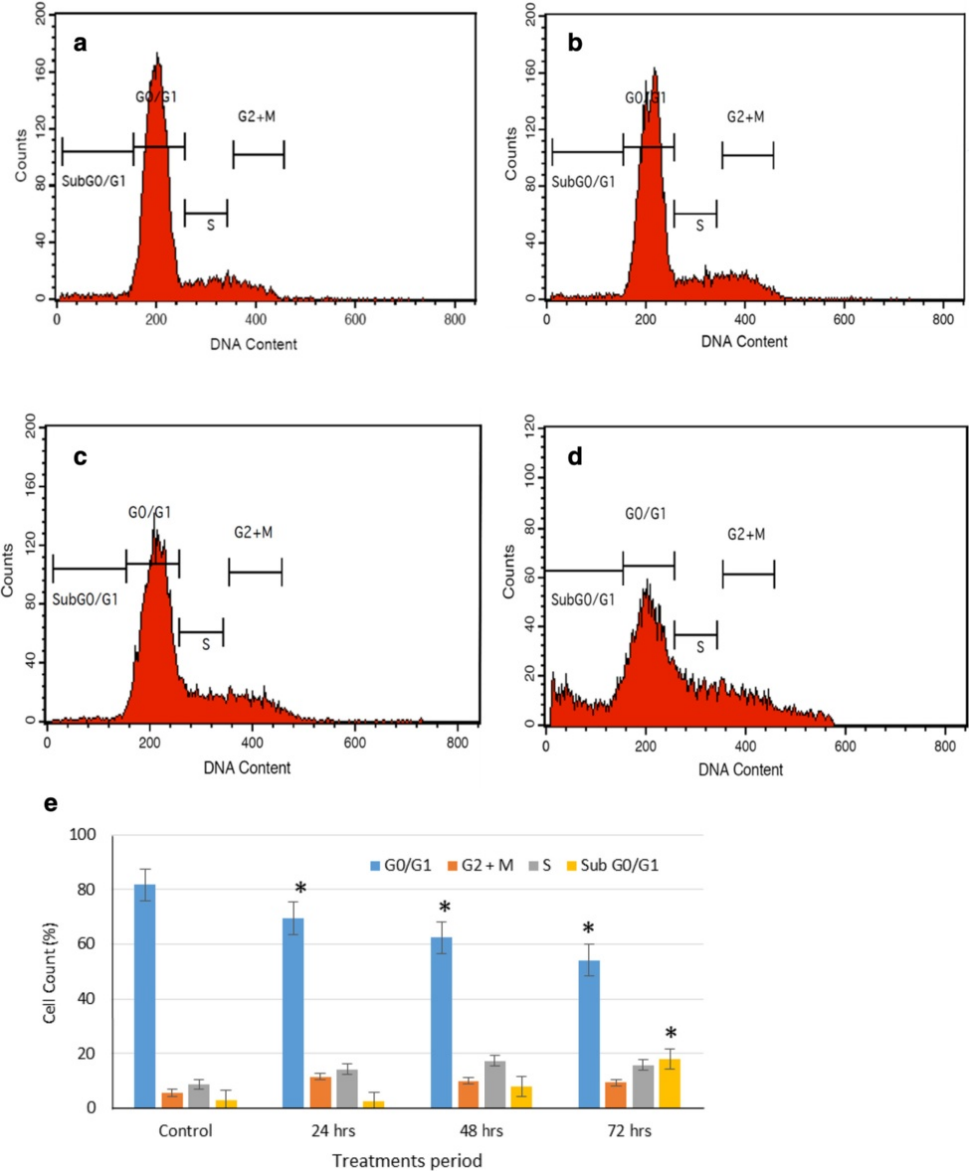
Cell cycle of clausenidin-treated HT-29 cells. **b**, **c**, **d** are DNA contents of cell treated for 24, 48, 72 h, respectively and (**a**) is untreated control cells. **e** represents % cell count at cell cycle phases. Analyses in triplicates. (*Significant difference between means at *p* < 0.05)

The DNA content histograms of HT-29 cells treated with clausenidin is shown in Fig. 7 which suggests a G0/G1 phase arrest. The percentage cell distribution (Fig. 7e) further reveals that clausenidin induces a G0/G1 phase arrest in HT-29 cells. The percentage of apoptotic cells (sub G0/G1) increased significantly (*p* < 0.05) compared to the control as treatment time progressed to 72 h (Fig. 7e) as a result of the cytotoxic effects of clausenidin.

### Annexin V Assay

**Fig. 8:**
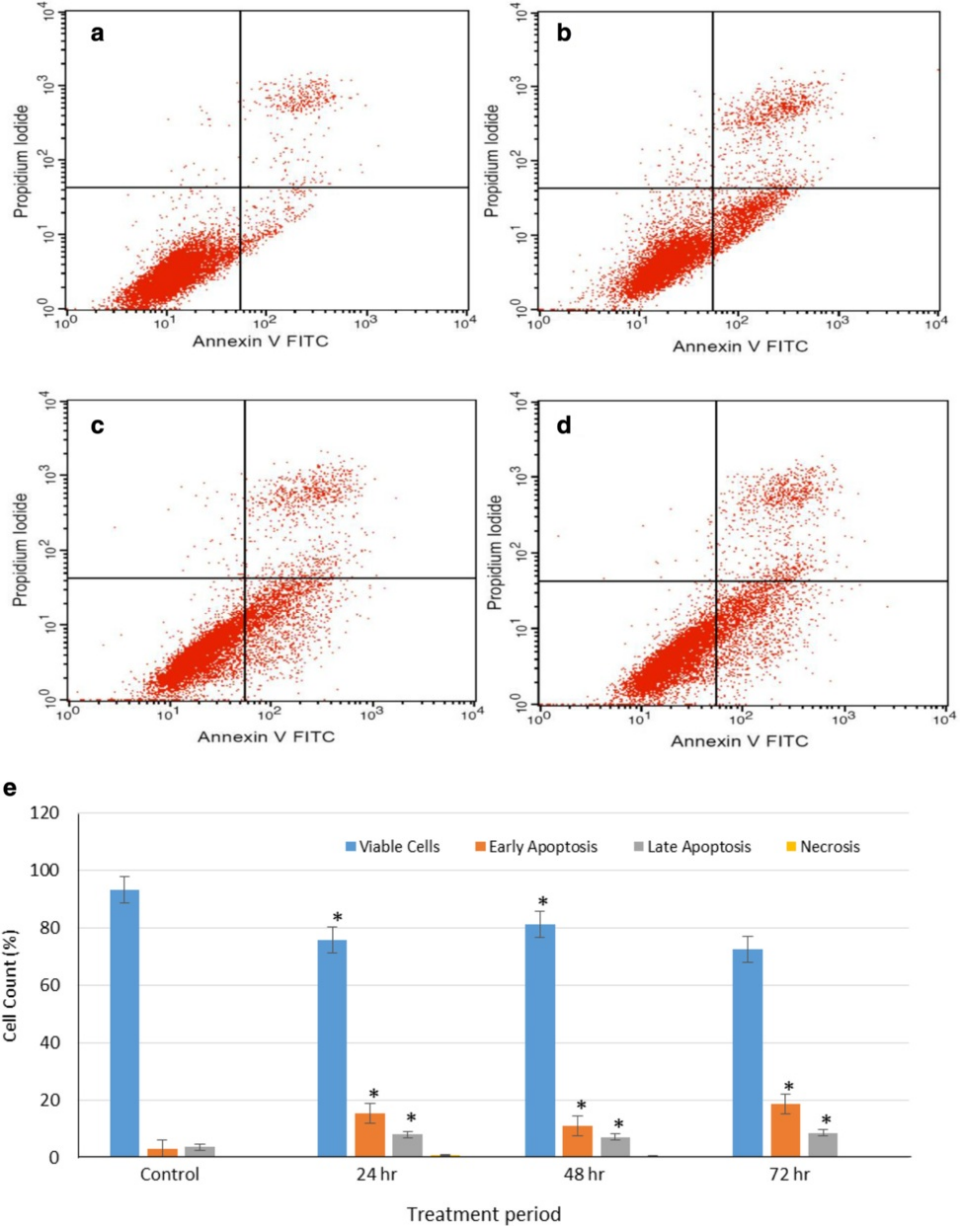
Annexin V assay of clausenidin-treated HT-29 cells. **b**, **c**, **d** represent viable and dead cells after treatment for 24, 48, 72 h, respectively and (**a**) is untreated control cells. **e** represents % cell count. Analyses in triplicates. (*Significant difference between means at *p* < 0.05)

The occurrence of apoptosis was further corroborated using the annexin V and PI staining assay because annexin V is able to bind membrane phospholipid which is released prior to the loss of membrane integrity. The annexin V assay distinguished between HT-29 cells in early and late apoptotic state after exposure to clausenidin (Fig. 8). Overall, the result in Fig. 8 shows a significant increase (*p* < 0.05) in the percentage of apoptotic cells resulting from clausenidin treatment in a time dependent manner compared to the untreated cells.

### MMP Assay

**Fig. 9:**
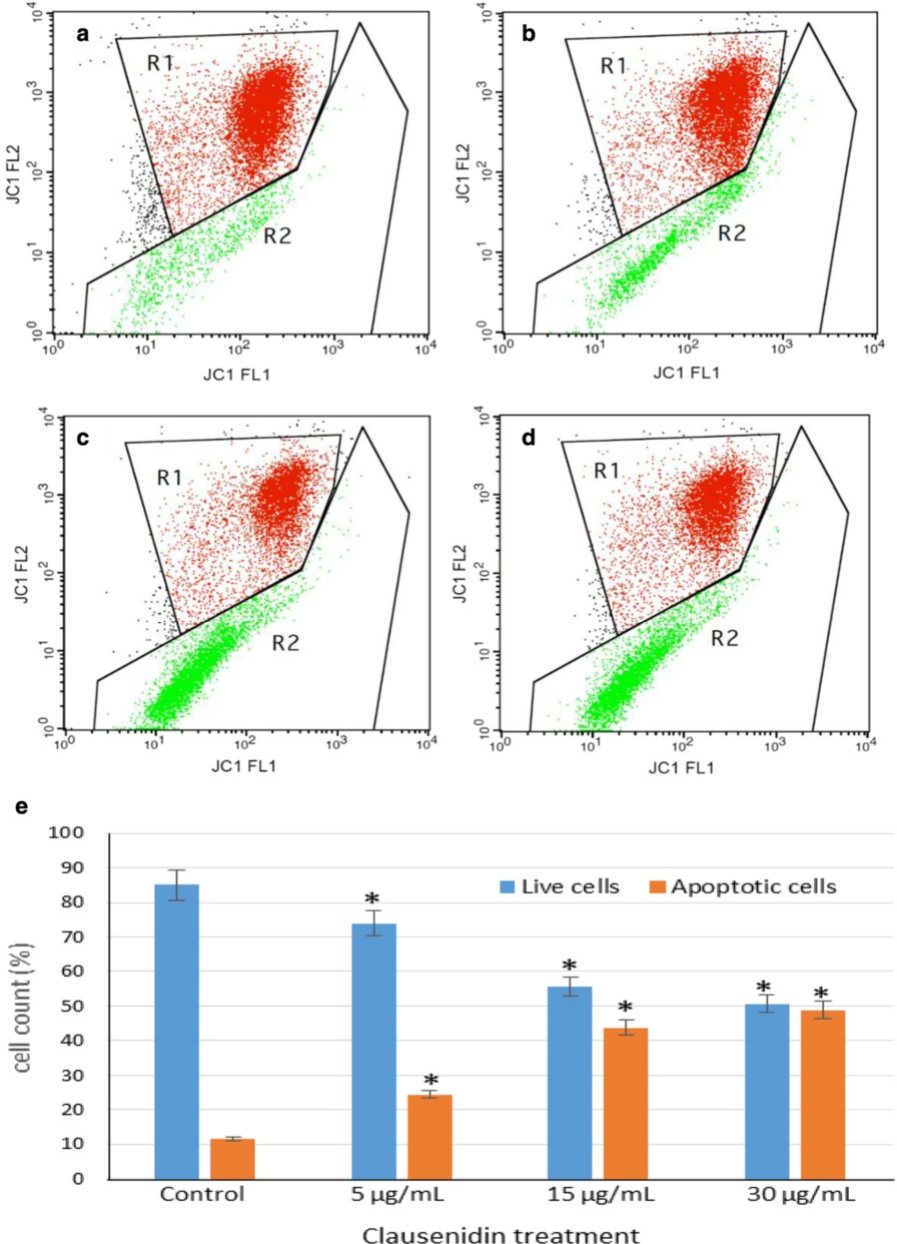
Mitochondrial membrane potential assay of clausenidin-treated HT-29cells. **b**, **c**, **d** represent treatment with 5, 15, and 30 μg/mL clausenidin respectively and (**a**) is the untreated control. Red fluorescence: cells with intact mitochondria, green fluorescence: cells with depolarized mitochondrial membrane. **e** represents proportion of live and apoptotic cells. Analyses in triplicates. (*Significant difference between means at *p* < 0.05)

The MMP assay was done to assess the mitochondrial function, since it is an organelle that is intimately involved in reception of signals that culminates in apoptosis. The MMP results shows a significant increase (*p* < 0.05) in collapse of MMP (green fluorescence) while a concomitant significant decrease (*p* < 0.05) was observed in the percentage of live cells with intact mitochondria (red fluorescence) (Fig. 9). The green fluorescence is as a result of increasing membrane depolarization while red fluorescence is a reflection of high membrane polarization after staining with the JC-1 dye.

### Caspases 3 & 9

**Fig. 10:**
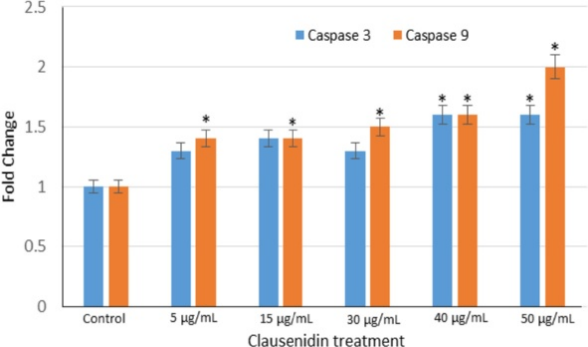
Caspase activities in clausenidin-treated HT-29 cells. Analyses in triplicates. (*Significant difference between means at *p* < 0.05)

Caspase 9 is an initiator caspase whose function enables the activation of executioner caspases such as caspase 3 [26]. The result in Fig. 10 shows clausenidin induces a significant increase (*p* < 0.05) in the expression of caspases 3 and 9 in HT-29 cells in a dose dependent manner.

### Gene expression studies

**Fig. 11:**
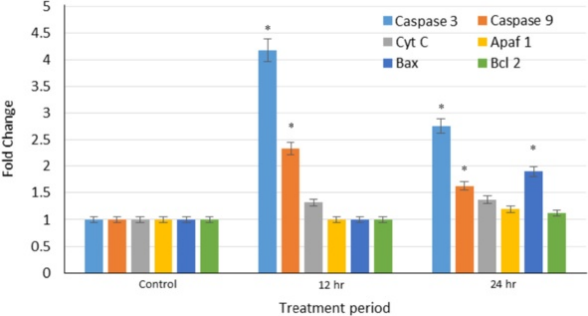
Caspase, Cyt c, Apaf 1, Bax and Bcl2 gene expressions in clausenidin-treated HT-29 cells. Analyses in triplicates. (*Significant difference between means at *p* < 0.05)

The gene expression studies was conducted to gain possible insights on how clausenidin affects the expression of caspases genes and other apoptotic genes associated with the mitochondrial pathway of apoptosis. The results shows that clausenidin significantly increases (*p* < 0.05) the expression of caspases 3 and 9 genes at 12 and 24 h of treatment (Fig. 11). Similarly, the expressions of Cyt c, Apaf-1, and Bax were significantly increased after 24 h of treatment with clausenidin compared to the untreated cells.

## Discussion

There are few literatures on the isolation of clausenidin and its potential cytotoxic effects but none of the reports have clearly defined the mechanism of action of clausenidin in any cancer cell line. In this particular study, we investigated the cascade of reactions that triggers apoptosis in HT-29 cells treated with clausenidin from *Clausena excavata*. It was observed that apoptosis occurred in the clausenidin treated HT29 cells via caspase 9 mediated signaling.

The purified clausenidin induced cytotoxic effects in HT-29 cells in a dose dependent manner with an IC_50_ of 13.8 ± 2.89 μg/mL. This could be a justification for the wide usage of *C excavata* in Asian folk medicine. Apoptosis is programmed active cell death. A number of anticancer drugs have been screened and selected based on their abilities to initiate the physiological events that culminates in cell death [7]. We observed the presence of membrane blebs, and chromatin condensation in the fluorescent micrographs of clausenidin treated HT-29 cells which represents features of incipient apoptosis [27]. Similarly, the ultrastructural micrograph revealed morphological aberrations within the organelles in HT-29 cells associated with apoptosis. The apoptotic features observed includes appearance of lipid droplets (as a result of cell membrane damage), condensation of chromatin and nuclear fragmentation which further corroborated apoptosis in the clausenidin treated HT-29 cells.

DNA fragmentation and loss of mitochondrial membrane integrity precedes apoptosis [8]. Clausenidin caused a nucleosomal DNA cleavage in HT-29 cells which led to the generation of DNA fragments as shown earlier in the gel image result. The generation of DNA fragments increased as the treatment time progressed suggesting the ability of clausenidin to sustain apoptosis in HT-29 cells. As a proof of the apoptosis-inducing effect of clausenidin, we observed a loss of MMP in the HT-29 cells which is a prerequisite for apoptosis to occur via the mitochondrial pathway. Also, apoptosis induced by some chemotherapeutic agents is controlled by the ratio of bax:bcl 2 expressions in the mitochondria [28]. Increased expression of Bax is known to stimulate a collapse of MMP which terminates in apoptosis [29]. Our gene expression study result shows a significant increase (*p* < 0.05) in the bax level which could have triggered the MMP collapse in clausenidin treated HT-29 cells observed earlier. In addition, a significant increase (*p* < 0.05) in cytochrome c and Apaf-1 genes expression were observed after treatment with clausenidin. Mitochondrial release of cytochrome c into the cytoplasm enables it to interact with dATP and Apaf-1 to form the apoptosome complex and subsequent activation of caspases [26]. The mitochondria has been described as a major component of the intrinsic pathway because of its ability to activate the apoptosis program directly. Therefore, we suspect that the increased levels of the apoptotic genes as observed in this study could have been triggered by clausenidin induced mitochondrial signaling. In a recent study with dentatin isolated also from *C. excavata*, it was observed that apoptosis occurred via mitochondrial pathway signaling in the dentatin-treated MCF-7 breast cancer cells [21].

Moreover, the expressions of caspases 3 and 9 proteins increased significantly (*p* < 0.05) after treating the HT-29 cells with clausenidin. Caspase 9 is a key regulator of mitochondrial apoptosis, because it functions as a gatekeeper of the intrinsic pathway [11]. So therefore, an increase in the expression of caspase 9 protein and gene as observed in this study strongly suggests the involvement of the intrinsic pathway (mitochondrial pathway) in the induction of apoptosis in clausenidin-treated HT-29 cells. Impaired functions of the caspase 9 promoter or caspase 9 gene which leads to low activity of caspase 9 has been implicated as a cause of various cancers such as colon cancer [30–33]. We observed that clausenidin treatment significantly increased (*p* < 0.05) the expression of caspase 9 gene by over two (2) folds. It is no surprise that the caspase 3 expression also increased significantly since it depends on caspase 9 for activation [11]. Caspase 3 executes apoptosis via selective destruction of subcellular structures, organelles or even the genome [11]. Our earlier results of TEM and DNA fragmentation analysis confirms this process of subcellular destruction. An increased expression of caspases 3 and 9 have also been reported in breast cancer cells treated with dentatin isolated from *C. excavata* [21].

One of the mechanisms by which anticancer agents induce apoptosis is through the creation of oxidative imbalance, which is a consequence of increased intracellular ROS production beyond the capacity of antioxidant defense system [34]. Previous studies have shown that there is a relationship between the mitochondrial derived ROS and the activation of caspases [35, 36]. The increased production of ROS in the present study could have triggered the clausenidin induced apoptosis in HT-29 cells as shown by our TEM micrographs. ROS has been reported to cause DNA strand cleavage as well as cell membrane injury [34] which we observed in the present study.

However, insensitivity to growth inhibitory signals has been proposed as one of the hallmarks of cancer survival strategy [11]. This leads to an inability to regulate the cell cycle which culminates in the development of cancer [37]. The cell cycle result shows that clausenidin induces a G0/G1 arrest in HT-29 cells. This finding could suggest another pathway through which clausenidin elicits signals that inhibits/controls the growth of tumor cells. Another vital observation in the cell cycle assay is the significant increase in the fractionated DNA of the clausenidin treated cells as represented by the sub G0/G1 fraction. This important finding and other results presented in this study lends credence to the occurrence of apoptosis in the clausenidin treated HT-29 cells.

## Conclusion

Apoptosis was induced successfully in colon cancer cells using clausenidin isolated from *C. excavata*. The clausenidin-stimulated apoptosis occurred via increased expressions of caspases 3 and 9. In addition, the current study justifies claims of anti-tumor properties of *Clausena excavata* Burm. *f*. in the traditional treatment of malignant cancers.

## Abbreviations

Apaf-1, Apoptotic protease activating factor-1; Bax, Bcl 2 associated x protein; Bcl 2, B cell lymphoma 2; Caspase, cysteine aspartic acid protease; Cyt c, Cytochrome complex; DMEM, Dulbecco’s Modified Eagle’s Medium; DMSO, Dimethyl sulfoxide; DNA, Deoxyribonucleic acid; HT-29 cells, Colon cancer cells; MTT, 3–(4,5-dimethylthiazol-2-yl)-2,5-Diphenyltetrazolium bromide; qPCR, quantitative Polymerase Chain reaction; ROS, Reactive oxygen species; TEM, Transmission electron microscopy.
